# Comprehensive Genome Analysis of Carbapenemase-Producing *Enterobacter* spp.: New Insights into Phylogeny, Population Structure, and Resistance Mechanisms

**DOI:** 10.1128/mBio.02093-16

**Published:** 2016-12-13

**Authors:** Kalyan D. Chavda, Liang Chen, Derrick E. Fouts, Granger Sutton, Lauren Brinkac, Stephen G. Jenkins, Robert A. Bonomo, Mark D. Adams, Barry N. Kreiswirth

**Affiliations:** a Public Health Research Institute Tuberculosis Center, New Jersey Medical School, Rutgers University – Newark, New Jersey, USA; b J. Craig Venter Institute (JCVI), Rockville, Maryland, USA; c Department of Pathology and Laboratory Medicine, Weill Cornell Medical Center, New York, New York, USA; d Research Service, Louis Stokes Veterans Affairs Medical Center, Cleveland, Ohio, USA; e J. Craig Venter Institute, La Jolla, California, USA

## Abstract

Knowledge regarding the genomic structure of *Enterobacter* spp., the second most prevalent carbapenemase-producing *Enterobacteriaceae*, remains limited. Here we sequenced 97 clinical *Enterobacter* species isolates that were both carbapenem susceptible and resistant from various geographic regions to decipher the molecular origins of carbapenem resistance and to understand the changing phylogeny of these emerging and drug-resistant pathogens. Of the carbapenem-resistant isolates, 30 possessed *bla*_KPC-2_, 40 had *bla*_KPC-3_, 2 had *bla*_KPC-4_, and 2 had *bla*_NDM-1_. Twenty-three isolates were carbapenem susceptible. Six genomes were sequenced to completion, and their sizes ranged from 4.6 to 5.1 Mbp. Phylogenomic analysis placed 96 of these genomes, 351 additional *Enterobacter* genomes downloaded from NCBI GenBank, and six newly sequenced type strains into 19 phylogenomic groups—18 groups (A to R) in the *Enterobacter cloacae* complex and *Enterobacter aerogenes*. Diverse mechanisms underlying the molecular evolutionary trajectory of these drug-resistant *Enterobacter* spp. were revealed, including the acquisition of an antibiotic resistance plasmid, followed by clonal spread, horizontal transfer of *bla*_KPC_-harboring plasmids between different phylogenomic groups, and repeated transposition of the *bla*_KPC_ gene among different plasmid backbones. Group A, which comprises multilocus sequence type 171 (ST171), was the most commonly identified (23% of isolates). Genomic analysis showed that ST171 isolates evolved from a common ancestor and formed two different major clusters; each acquiring unique *bla*_KPC_-harboring plasmids, followed by clonal expansion. The data presented here represent the first comprehensive study of phylogenomic interrogation and the relationship between antibiotic resistance and plasmid discrimination among carbapenem-resistant *Enterobacter* spp., demonstrating the genetic diversity and complexity of the molecular mechanisms driving antibiotic resistance in this genus.

## IMPORTANCE

*Enterobacter* spp., especially carbapenemase-producing *Enterobacter* spp., have emerged as a clinically significant cause of nosocomial infections. However, only limited information is available on the distribution of carbapenem resistance across this genus. Augmenting this problem is an erroneous identification of *Enterobacter* strains because of ambiguous typing methods and imprecise taxonomy. In this study, we used a whole-genome-based comparative phylogenetic approach to (i) revisit and redefine the genus *Enterobacter* and (ii) unravel the emergence and evolution of the *Klebsiella pneumoniae* carbapenemase-harboring *Enterobacter* spp. Using genomic analysis of 447 sequenced strains, we developed an improved understanding of the species designations within this complex genus and identified the diverse mechanisms driving the molecular evolution of carbapenem resistance. The findings in this study provide a solid genomic framework that will serve as an important resource in the future development of molecular diagnostics and in supporting drug discovery programs.

## INTRODUCTION

In the last decade, the emergence of carbapenem resistance in *Enterobacteriaceae* became a significant public health concern, as carbapenems are regarded as being among the few antibiotics that can be used to treat severe infection in this family of common bacterial pathogens. Dissemination of carbapenemase-producing *Enterobacteriaceae* (CPE) in the United States has largely been associated with the class A β-lactamase *Klebsiella pneumoniae* carbapenemase (KPC). The resistance gene, *bla*_KPC_, is typically plasmid borne and can move between genera of *Enterobacteriaceae*, thereby facilitating its dissemination (1).

The first KPC-producing strain identified in the United States was a *K. pneumoniae* isolate collected in a North Carolina hospital in 1996 (2). Since then, KPC-producing isolates have spread globally and into various Gram-negative species (predominantly in *K. pneumoniae*); with KPC outbreaks reported in New Jersey and New York City hospitals (2–7). Much like the emergence and epidemic spread of carbapenem-resistant *K. pneumoniae* in the New York-New Jersey region, *bla*_KPC_-harboring isolates among other members of the family *Enterobacteriaceae*, especially *Enterobacter* spp., were frequently identified in clinical settings, representing a major infection control and therapeutic challenge. Overall, *Enterobacter* spp. are the sixth leading cause of health care-associated infections globally (8).

The first strain of an *Enterobacter* species carrying plasmid-encoded *bla*_KPC-2_ was obtained from a patient with sepsis at a Boston hospital in 2001 (9). Since its initial identification, sporadic cases with KPC-harboring *Enterobacter* spp. have been described and several outbreaks have been reported worldwide (10–15). According to two surveillance studies (in 2006 and 2009), a total of 758 KPC-positive Gram-negative isolates were collected, revealing that *Enterobacter* spp. were second to *K. pneumoniae* in harboring the *bla*_KPC_ gene (16). In a study in Detroit between September 2008 and September 2009, *bla*_KPC_-harboring *Enterobacter* spp. accounted for ~15% of the carbapenem-resistant *Enterobacteriaceae* isolates in an urban health care system (17). Other studies have reported the spread of carbapenem resistance plasmids among related yet polyclonal strains of phenotypically similar *Enterobacter* spp. (11, 18), while another report describes the clonal dissemination of an outbreak strain between hospitals (19).

Despite the clinical significance of KPC-harboring *Enterobacter* spp., little attention has been focused on understanding the evolution and spread of *bla*_KPC_ in *Enterobacter* spp. The molecular epidemiology of these strains in terms of genetic background, *bla*_KPC_ type, and the structure of Tn*4401* and nature of the plasmids harboring these resistant transposons, remains unknown. With this in mind, we sequenced to completion the genomes of six different KPC-producing *Enterobacter* spp. clinical isolates and then performed comparative whole-genome sequencing of 91 additional genomes of diverse *Enterobacter* clinical isolates obtained from the United States, South America, and the Mediterranean region to define the genetic structure of these drug-resistant emerging pathogens. These sequences were placed in a broader phylogenetic context by including 351 publicly available *Enterobacter* genome sequences in our analysis, including 77 that carry the *bla*_KPC_ gene, and by performing complete genome sequencing of an additional six *Enterobacter* type strains.

## RESULTS

### Complete sequencing of six clinical isolates of carbapenem-resistant *Enterobacter* spp.

**FIG 1:**
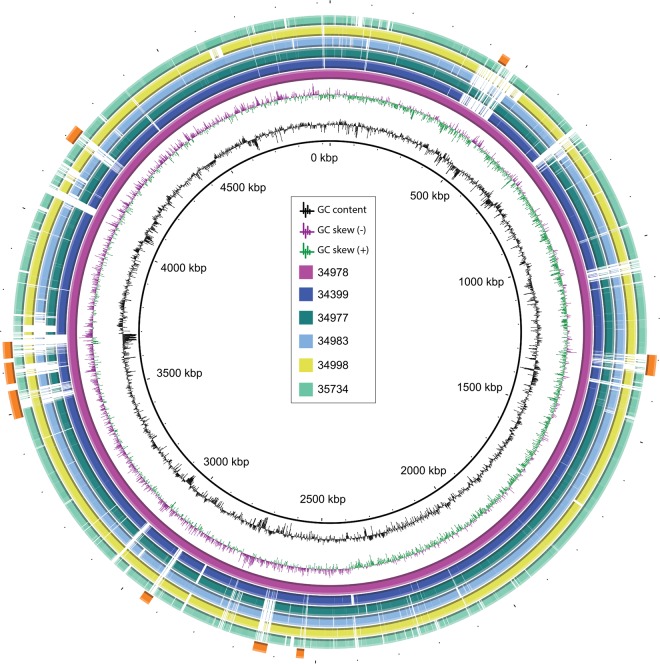
*Enterobacter* sp. genomes. Five completely sequenced *Enterobacter* genomes were compared with the *E. hormaechei* ST171 strain 34978 genome (pink inner circle). Genes that are present in 34978 but not in the other genomes are shown as blank spaces in the rings representing the five genomes. The GC content and GC skew of strain 34978 are plotted, and prophage locations are indicated by orange rectangles.

In this study, we sequenced to closure six *bla*_KPC_-harboring *Enterobacter* spp. clinical isolates by using Pacific Biosciences (PacBio) single-molecule real-time sequencing technology. The six isolates were obtained between 2011 and 2012 from patients at different health care institutions in New York, Florida, and Illinois (Table 1). Their genome sizes ranged from 4.6 to 5.1 Mbp (Fig. 1), similar in length to other completely sequenced *Enterobacter* spp. genomes (range, 4.5 to 5.4 Mbp) (20–23). *In silico* multilocus sequence typing analysis of seven target genes (*dnaA*, *fusA*, *gyrB*, *leuS*, *pyrG*, *rplB*, and *rpoB*) developed by Miyoshi-Akiyama et al. (24) assigned the six isolates to multiple sequence types (STs), ST113, ST114, ST171, ST269, ST594 and ST595. These isolates contained 4,275 to 4,559 coding genes and harbored between three and nine prophages (Table 1). The average GC content of the chromosomes was 55.41%, with a minimum and a maximum of 55.1% for BK34978 and 55.8% for BK35734. Moreover, these isolates harbored 83 or 84 tRNA genes and 25 rRNA genes.

**TABLE 1:** Key features of six clinical *Enterobacter* isolates sequenced to closure

Characteristic	34399	34977	34978	34983	34998	35734
Species or subspecies	*E. xiangfangensis*	*E. hormaechei* subsp. *steigerwaltii*	*E. xiangfangensis*	*E. hormaechei* subsp. *hormaechei*	*E. hormaechei* subsp. *steigerwaltii*	*E. cloacae* complex Hoffmann cluster IV
ST	114	594	171	269	113	595
Location	Illinois	New York City	New York City	New York City	New York City	Florida
Phylogenetic group	A	B	A	E	B	M
Resistance gene(s)						
Chromosome	*bla*_ACT-25_	*bla*_ACT-42_, *aph*(*3*′)*-Ia*	*bla*_ACT-45_, *aph*(*3*′)*-Ia*	*bla*_ACT-37_	*bla*_ACT-32_	*bla*_MIR-20_, *qnrB19*, *qnrB19*
Plasmids	*qnrS1*, *bla*_KPC-3_, *bla*_TEM-1A_	*bla*_KPC-2_, *bla*_TEM-1B_, *bla*_SHV-12_, *strB*, *strA*, *aadA2*, *aac*(*6.0*′)*-Iic*, *aph*_(*3*_′_)_*-Ia*, *qnrB2*, *ere*_(*A*)_, *sul1*, *sul1*, *sul1*, *sul2*, *dfrA18*	*bla*_KPC-3_, *bla*_OXA-9_, *bla*_TEM-1A_, *aac*(*6.0*′)*-Ib*, *aadA1*, *strB*, *strA*, *aac*(*6.0*′)*-Ib-cr*, *sul2*, *dfrA14*	*bla*_KPC-2_, *bla*_TEM-1B_, *qnrB2*, *sul1*, *sul1*, *dfrB3*	*bla*_KPC-4_, *bla*_TEM-1A_, *bla*_OXA-1_, *aadA1*, *aac*(*3*)*-via*, *aph*_(*3*_′_)_*-Ic*, *aac*(*6.0*′)*-Ib-cr*, *aac*(*6.0*′)*-Ib-cr*, *mph*(*A*), *catB3*, *arr-3*, *sul1*, *sul1*, *strA*, *strB*, *bla*_TEM-1B_, *sul2*, *tet*_(*D*)_, *dfrA14*, *qnrS1*	*bla*_KPC-3_, *bla*_KPC-3_, *bla*_OXA-9_, *bla*_TEM-1A_, *aac*(*6.0*′)*-Ib*, *aadA1*, *aac*(*6.0*′)*-Ib-cr*, *qnrB19*
Genome size (bp)	4,784,288	4,897,485	4,930,963	4,632,074	4,688,189	5,017,289
% G+C	55.3	55.4	55.1	55.2	55.7	55.8
No. of coding sequences	4458	4495	4559	4275	4310	4493
No. of plasmids	3	3	6	3	5	3
No. of prophages	7	3	9	5	6	8
No. of IS elements	6	12	17	35	5	31
No. of tRNA genes	84	83	83	84	84	83
No. of rRNA genes	25	25	25	25	25	25

All six strains were resistant to β-lactam antibiotics (including carbapenem antibiotics), except BK34983, which has intermediate susceptibility to imipenem and meropenem but was susceptible to doripenem (see Data Set S1 in the supplemental material). Moreover, they were resistant to β-lactamase inhibitors (e.g., clavulanate, sulbactam, and tazobactam) and showed variable resistance to quinolones (ciprofloxacin, moxifloxacin, and levofloxacin), aminoglycosides (amikacin, gentamicin, and tobramycin), and tetracycline (tetracycline, doxycycline, and minocycline) (see Data Set S1). The majority of the antibiotic resistance determinants described above were located on plasmids (Table 1; see Table S1 in the supplemental material). These strains contained three to six plasmids, varying in size from 2.7 to 328 kbp, that collectively encode resistance to multiple classes of antibiotics (see Table S1).

In four of the six strains, *bla*_KPC_-harboring plasmids (p34977-B, p34983-A, p34399-A, and p35734-B) were identical or highly similar to conjugative plasmid pKPC_UVA01, initially described from *K. pneumoniae* isolates recovered from patients in the University of Virginia Health System and long-term acute care hospital (see Fig. S1 in the supplemental material) (25). *bla*_KPC_-harboring Tn*4401* in this plasmid was inserted within the *tnpA* gene of a Tn*2*-like element. Identical to pKPC_UVA01, plasmids p34977-B and p34983-A were 43,621 bp in size, carried a novel replicon (nontypeable by current incompatibility [Inc] group typing) (26), and harbored *bla*_KPC-2_ on a Tn*4401b-*Tn*2*-like transposon structure. In comparison to pKPC_UVA01, the *bla*_KPC-3_-harboring Tn*4401b*-Tn*2*-like structure in p34399-A was incorporated at a distinct location between genes encoding a hypothetical protein and a chaperonin protein, demonstrating independent acquisition of *bla*_KPC_ in the same plasmid backbone. Moreover, a similar pKPC_UVA01-like plasmid (pKPC-f91) was found in strain ECNIH2, isolated from a sink drain at the National Institutes of Health (NIH) Clinical Center (see Fig. S1) (21). Interestingly, p35734-B is similar to p34399-A with an insertion of another plasmid sequence related to plasmid pENT-c88 from strain ECNIH4 (21). Plasmid p35734-B harbored two copies of *bla*_KPC-3_ on two separate Tn*4401b* elements; however, *bla*_KPC_ was not located within the Tn*4401*-Tn*2*-like structure as described above. The potential functional significance of this duplication appears to be supported by the elevated MICs of carbapenem antibiotics for strain BK35734 (see Data Set S1).

Plasmid p34978-F is identical to IncFIA plasmid pBK30683 from *K. pneumoniae* (27), which is one of the most predominant *bla*_KPC_-harboring plasmids circulating in *K. pneumoniae* strains in New York-New Jersey hospitals (see Table S1). Of note, pBK30683 is a cointegrate plasmid that has an ~70-kb IncFIA plasmid backbone nearly identical to that of pBK30661 and the integration of the second IncF plasmid harboring the *tra* operon for plasmid transfer (27). Therefore, IncFIA pBK30683-like plasmids have two different forms, the nonconjugative pBK30661 type and the cointegrate pBK30683 type (pBK30661 containing a second IncF plasmid carrying the functional *tra* operon) (27).

Interestingly, strain BK34998 has a *bla*_KPC-4_-harboring IncA/C plasmid, p34998-E. p34998-E carries a plasmid backbone similar to that of the other IncA/C plasmids pRA1 (28) (IncA/C reference plasmid isolated in 1971 from *Aeromonas hydrophila*) and p35734-C (isolated from strain BK35734) (see Fig. S2 in the supplemental material). However, p34998-E acquired an ~66-kb region encoding genes for β-lactam resistance, aminoglycoside resistance, quinolone resistance, phenicol resistance, macrolide resistance, sulfonamide resistance, and rifampin resistance (see Fig. S2). The region surrounding *bla*_KPC-4_ showed homology to the *bla*_KPC-4_ region from IncN plasmid pBK31551 from *K. pneumoniae* that we described previously (29).

In addition to the *bla*_KPC_-harboring plasmids, the six PacBio sequenced strains harbored an additional two to five plasmids. These plasmids ranged in size from 2,725 to 328,905 bp, and most of them did not carry any antimicrobial resistance genes (see Table S1). Among them, p34977-A; p34978-A, -B, -C, and -D; p34998-A; and p35734-A belonged to the ColE superfamily. The two IncF plasmids p34399-B (from 34399, ST114) and p34998-C (from 34998, ST113) were nearly identical and both harbored the quinolone resistance gene *qnrS1* (see Table S1). In contrast, plasmids p34983-B and p34998-B belonged to the same incompatibility group, IncN3, but share only 51% homology with 97% identity. Plasmid p34977-C is an IncHI2 plasmid and harbors genes for resistance to β-lactams (*bla*_SHV-12_), aminoglycosides [*strB*, *strA*, *aadA2*, *aac*(*6*′)*-IIc*, *aph*(*3*′)*-Ia*], quinolones (*qnrB2*), sulfonamide (*sul1*, *sul2*), trimethoprim (*dfrA18*), and macrolides [*ere*_(*A*)_]. Plasmid p34983-C belongs to the IncHI1 group but does not harbor any antimicrobial resistance genes. Lastly, plasmid p35734-C belongs to the IncA/C group and shares homology with *bla*_KPC-4_-carrying plasmid p34998-E, as described above. However, p35734-C does not harbor any resistance genes (see Fig. S2).

### Genome sequencing of geographically diverse *Enterobacter* spp. clinical isolates.

To gain a better understanding of the emergence and evolution of the *bla*_KPC_-harboring *Enterobacter* spp. and to perform a whole-genome-based phylogenetic classification of these isolates, we next performed whole-genome sequencing of 91 additional *Enterobacter* clinical isolates (see Data Set S1). Among them, 66 possessed *bla*_KPC_, 2 had *bla*_NDM_, and 23 were carbapenem susceptible. Isolates were selected on the basis of their geographic and temporal distributions, KPC variants, and *Tn*4401 patterns. Isolates were obtained from health care facilities at diverse geographic locations in the United States (Florida, Illinois, Michigan, Ohio, Pennsylvania, New Jersey, New York, and Texas), Gaza, and Colombia between 2006 and 2014. Strain 40874 was identified as *Pluralibacter gergoviae* (formerly *E. gergoviae*) and was not analyzed further.

### Phylogenetic structure of *Enterobacter* species.

To estimate the genetic relationships among *Enterobacter* strains, the 97 genomes sequenced in this study were compared with 351 *Enterobacter* genomes publicly available in GenBank by using both average nucleotide identity (ANI) and a single-nucleotide polymorphism (SNP)-based phylogeny. SNPs were identified from the combined set of genome sequences by using kSNP (30). Nucleotide positions present in at least 80% of all genomes were used to build a phylogenetic tree with RAxML (31).

**FIG 2:**
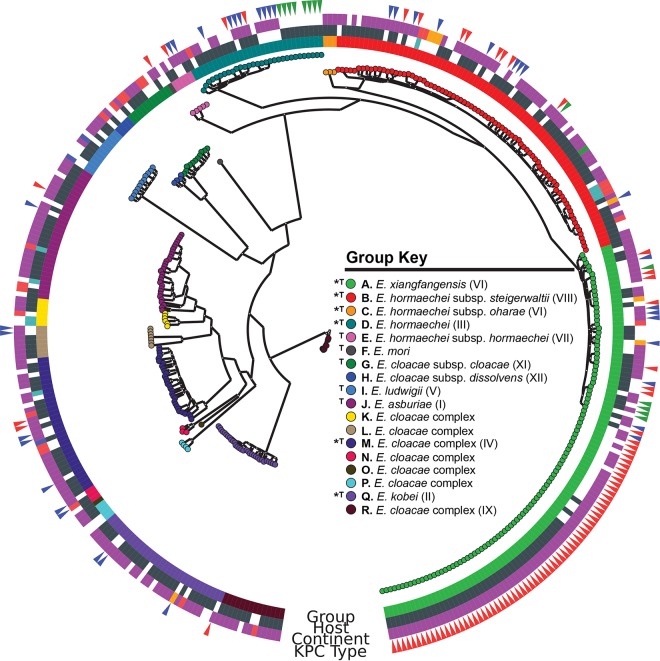
Phylogenetic SNP tree of *E. cloacae* complex genomes. A whole-genome core SNP tree was constructed for 379 *E. cloacae* complex genomes with kSNP (30) and RAxML (31) (see Materials and Methods). Groups identified by ANI (A to R) are noted as colored nodes, as well as the innermost circle (see group key). Hosts, continents of origin, and KPC type metadata are also plotted in concentric circles as noted. Host colors: human, dark gray; nonhuman, blue. Continent colors: North America, purple; South America, orange; Australia, green; Europe, blue; Asia, red. KPC type colors: 3, red; 2, blue; 4, green.

Strains were clustered into 19 phylogenetic groups. One group corresponded to *E. aerogenes*, and the remaining 18 groups belong to the *E. cloacae* complex (groups A to R in Fig. 2; Table 2; see Fig. S4 and Data Sets S1 and S2 in the supplemental material) (32–34). The 18 *E. cloacae* complex groups typically had mean within-group ANI values >99% but always >96.5%. Mean ANI values between groups were always ≤95%, except among *E. hormaechei* subspecies groups A to E, G, and H (Table 2). Groups J and K may turn out to be subspecies, since they have 95% ANI. ANI and SNP phylogeny were concordant in clustering the genomes into phylogenetic groups. Additional details regarding the recommended ANI threshold to define species groups are described in Text S1 in the supplemental material.

**TABLE 2:** Group labels for the *E. cloacae* complex

Group	Hoffman cluster	Species, subspecies, or complex	Type strain	Accession no.	No. of strains
A	VI	*E. xiangfangensis*	LMG 27195^a^	NZ_CP017183.1	3
B	VIII	*E. hormaechei* subsp. *steigerwaltii*	DSM 16691^a^	NZ_CP017179.1	83
C	VI	*E. hormaechei* subsp. *oharae*	DSM 16687^a^	NZ_CP017180.1	104
D	III	*E. hormaechei*	DSM 14563^a^	NZ_CP017186.1	30
E	VII	*E. hormaechei* subsp. *hormaechei*	ATCC 49162	GCA_000213995.1	5
F		*E. mori*	LMG 25706	AEXB00000000	1
G	XI	*E. cloacae* subsp. *cloacae*	ATCC 13047	NC_014121.1	11
H	XII	*E. cloacae* subsp. *dissolvens*			4
I	V	*E. ludwigii*	EN-119	CP017279.1	11
J	I	*E. asburiae*	ATCC 35953	NZ_CP011863.1	30
K		*E. cloacae* complex			6
L		*E. cloacae* complex			7
M	IV	*E. cloacae* complex	DSM 16690^a^	NZ_CP017184.1	31
N		*E. cloacae* complex			3
O		*E. cloacae* complex			1
P		*E. cloacae* complex			4
Q	II	*E. kobei*	DSM 13645^a^	NZ_CP017181.1	31
R	IX	*E. cloacae* complex			14

a Type strains sequenced in this study.

### Identifying members of the *E. cloacae* complex to the subspecies level.

Before the advent of routine genome sequencing of type strains for validly published bacterial taxonomic species, there was quite a bit of confusion over what species make up the *E. cloacae* complex, with various species being moved to or from other genera or being reassigned as subspecies. Furthermore, many genomes have been submitted to GenBank as *E. cloacae* when they were in the *E. cloacae* complex but not the *E. cloacae* species, causing more confusion.

In a seminal work, Hoffmann and Roggenkamp (32) defined 12 genetic clusters (I to XII) based most exhaustively on *hsp60* sequencing. Three of the clusters (cluster III, 58 strains; cluster VI, 28 strains; cluster VIII, 59 strains) accounted for 70% of the 206 strains studied. In a following study, Hoffmann et al. (35) named cluster VII *E. hormaechei* subsp. *hormaechei*, cluster VI *E. hormaechei* subsp. *oharae*, and cluster VIII *E. hormaechei* subsp. *steigerwaltii*. More recently, Gu et al. (36) defined *Enterobacter xiangfangensis* by using a phylogenetic tree based upon concatenated partial *rpoB*, *atpD*, *gyrB*, and *infB* gene sequences from a novel isolate and existing type strains where *E. xiangfangensis* was closest to *E. hormaechei* in the tree. Details of the previous taxonomic work are described in Text S1 in the supplemental material.

In order to clarify the proper naming of genomes in the *E. cloacae* complex, we sequenced the type strains for *E. hormaechei* subsp. *steigerwaltii* (B; DSM 16691), *E. hormaechei* subsp. *oharae* (C; DSM 16687), *E. xiangfangensis* (A; LMG 27195), Hoffmann cluster III (D; DSM 14563), Hoffmann cluster IV (M; DSM 16690), and *E. kobei* (Q; DSM 13645) (Table 2).

To link our ANI and kSNP trees to the *hsp60* typing results, we performed “*in silico*” *hsp60* typing of the genome sequences and included previously published *hsp60* sequences. The *hsp60* types for species type strains, species type strains with sequenced genomes, and ANI values within and between groups were used to assign names to groups A to R (Table 2). Genomes from type strains were not available for all groups, so the name designations should be considered provisional. It should be noted that group D (*hsp60* cluster III) was not treated by Hoffmann as an *E. hormaechei* subspecies on the basis of *hsp60* sequence clustering results but by ANI represents a novel *E. hormaechei* subspecies. Group A, which includes ST171 strains and the type strain of *E. xiangfangensis*, also is an *E. hormaechei* subspecies based on ANI analysis. Groups G and H have a mean between-group ANI of 95.7%, which is consistent with having different *hsp60* types and being defined as different *E. cloacae* subspecies (35).

### Relationship of *E. aerogenes* to other members of the family *Enterobacteriaceae*.

**FIG 3:**
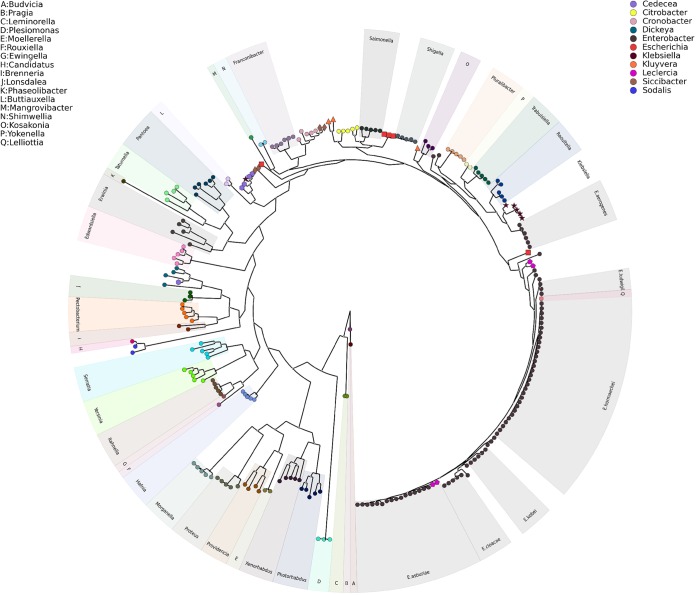
Phylogenetic universal marker tree of representative genomes of members of the family *Enterobacteriaceae*. A maximum-likelihood tree was constructed from a concatenated alignment of 26 conserved universal marker alleles from 248 genomes belonging to 48 different genera. If a genus was represented by one to three genomes, the genus was given a letter designation (A to Q; see key, top left). If a genus was represented by four or more genomes and all members clustered together, the genus was labeled directly on the tree; however, if they did not all cluster together, unique noncircular shapes were used to identify them. Each genus is assigned a unique color (see key, top right).

The group of *E. aerogenes* genomes was distant from other groups that represent the *E. cloacae* complex (e.g., *E. hormaechei*, *E. kobei*, *E. cloacae*, and *E. asburiae*). To better place the *E. aerogenes* genomes in the context of other members of the family *Enterobacteriaceae*, genomic sequences of 248 bacterial strains from 48 genera within the *Enterobacteriaceae* family were downloaded from GenBank and used to construct a phylogenetic tree (Fig. 3). It is apparent from the tree that *E. aerogenes* is more closely related to *K. pneumoniae* than to the *E. cloacae* complex (23). This is consistent with previous findings and proposals to rename the species: *K. mobilis* (37–39), *K. aeromobilis* (23), and very recently *K. aerogenes* (40).

### Core pangenome of *E. hormaechei*.

**FIG 4:**
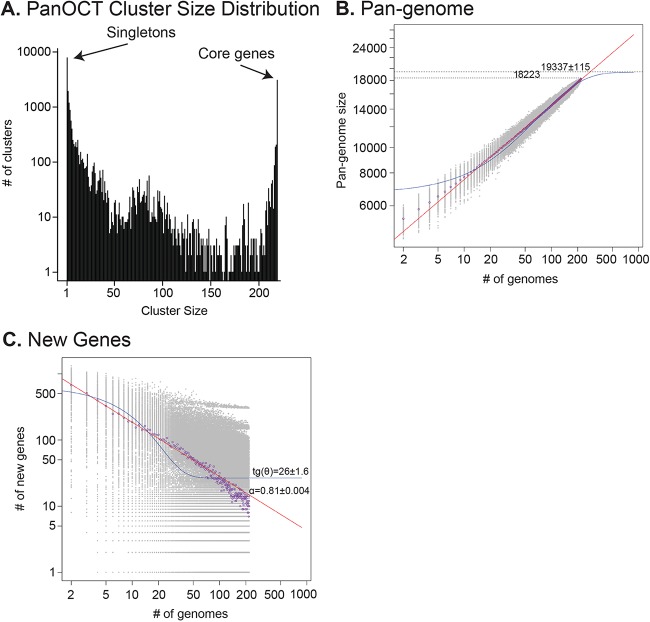
Analysis of the *E. hormaechei* pangenome. The distribution of protein cluster sizes (the number of genomes sharing each ortholog) generated from the comparison of 219 *E. hormaechei* genomes with PanOCT (42) indicates the numbers of singleton and core genes (A). The pangenome size (B) and the number of novel genes discovered with the addition of each new genome (C) were estimated by using a pangenome model as described previously (44). Purple circles are the median of each distribution (gray circles). Power law (red lines) and exponential (blue lines) regressions were plotted to determine α (open/closed status) and tg(θ), the average extrapolated number of strain-specific/novel genes, respectively (45).

To explore the potential functional significance of distinct *E. hormaechei* subspecies, the pangenome of all available *E. hormaechei* genomes was determined (groups A to E, as defined above) with the PanOCT software suite (41, 42). When the core pangenome of all 219 genomes analyzed (100%) was defined, there were 3,048 core/universal protein clusters and 7,794 singleton clusters (i.e., clusters with a single member from a single genome) identified (Fig. 4a). If the core pangenome were instead defined as clusters having protein members from 95% of the genomes analyzed, the core pangenome was 3,718 protein clusters. The pangenome of *E. hormaechei* appeared to be open (α = 0.8182 ± 0.004; Fig. 4b). The number of new genes found for each genome added to the pangenome was determined from the exponential decay function to be 26 ± 1.6 (Fig. 4c). Details of the analysis of the *E. hormaechei* pangenome are described in Text S1 in the supplemental material.

### fGIs define phenotypic differences between *E. hormaechei* subspecies.

**FIG 5:**
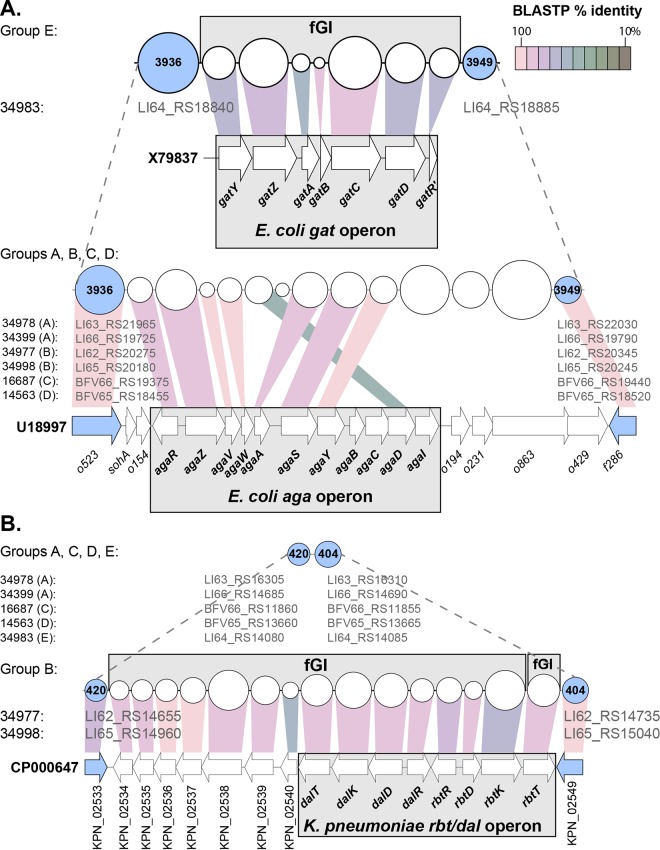
Historical metabolic differences defining *E. hormaechei* subspecies explained by fGI content. Variability within the flexible genomic region between core pangenome gene clusters 3936 and 3949 can contain either the *gat* operon to metabolize galactitol (defining group E) or the *aga* operon to metabolize *N*-acetylgalactosamine (defining groups A to D) (A). The presence of an operon between core gene clusters 420 and 404 homologous to the *Klebsiella rbt/dal* operon (75), for d-arabinitol and ribitiol catabolism, distinguishes group B isolates from A, C, D, and E isolates (B). Circles and arrows represent PanOCT gene clusters and protein coding regions, respectively. The size of a circle is scaled to the gene length of its centroid. The locus identifiers of core genes from applicable PacBio genomes in this study are noted in gray under their respective core clusters. Colored connecting lines represent the levels of conservation between those cluster centroids and genes whose protein sequences match at a BLASTP identity of ≥35% (see key, upper right). Blue circles and arrows denote flanking core clusters and genes, respectively.

*E. hormaechei* subsp. *hormaechei* (*hsp60* cluster VII, group E) is distinguishable from *E. hormaechei* subsp. *oharae* (*hsp60* cluster VI, group C) and *E. hormaechei* subsp. *steigerwaltii* (*hsp60* cluster VIII, group B) by growing on dulcitol (also called galactitol) as the sole carbon source (33). This phenotype of group E can be explained by the presence of a *gat* operon (43) located on a flexible genomic island (fGI) between core gene clusters 3936 (d-galactarate dehydratase) and 3949 (16S rRNA methyltransferase) (Fig. 5A), while at the same location, the *E. hormaechei* subspecies in groups A to D have a related but different operon, encoding *N*-acetylgalactosamine metabolism (also called the *aga* locus) (44) (Fig. 5A). Similarly, *hsp60* cluster VIII (B) isolates can be distinguished from *hsp60* cluster VI and VII isolates (A, C, D, and E) by their ability to grow on adonitol (also called ribitol) and d(+)-arabitol, both five-carbon sugar alcohols known as penitols. An fGI was identified that encodes the *rbt* and *dal* operons, which metabolize ribitol and d(+)-arabitol, respectively (45) (Fig. 5B).

### Plasmid gene content can influence the topology of the gene content tree.

From the PanOCT output, we can draw trees based on gene content. The gene content tree should be congruent with trees generated from analyses that favor vertical conservation (e.g., kSNP, universal markers, ANI), unless there has been recent acquisition of genes via lateral transfer. A tree generated from the PanOCT ortholog gene clusters representing the presence/absence of genes in each genome (see Fig. S3A in the supplemental material) showed a different clustering of genomes than was present in the kSNP phylogenic tree (Fig. 2). When ortholog clusters corresponding to genes that matched the 34 known plasmids (harbored by the complete genomes used in the PanOCT analysis) were removed, the topology of the tree resembled the kSNP tree more closely (see Fig. S3B).

### Plasmid diversity among *Enterobacter* spp.

The *bla*_KPC_ gene is reported to be found on plasmids of different Inc groups. In previous studies, certain *bla*_KPC_-harboring plasmids were found to be more prevalent among CPE isolates in New York-New Jersey hospitals, including the pBK30683/pBK30661 (IncFIA group) (27), pBK15692 (IncI2 group) (46), and pKpQIL (IncF_IIK2_ group) (47) plasmids. The plasmid sequences identified from the six closed PacBio genomes suggest that some plasmids found in *K. pneumoniae* are common in *Enterobacter* spp. For example, the *bla*_KPC_-harboring plasmid in 34978, p34978-F, is identical to IncFIA plasmid pBK30683 in *K. pneumoniae*. In addition, in four closed genomes from different phylogenetic groups, *bla*_KPC_ was found to be located on the same pKPC_UVA01-like plasmid. To estimate the occurrence of different *bla*_KPC_-harboring plasmids among the 72 KPC-producing *Enterobacter* strains, we mined the *bla*_KPC_-bearing Tn*4401* isoforms and their target site duplication (TSD) sequences and examined each *bla*_KPC_-harboring contig with BLASTn against completely sequenced plasmids in GenBank. Tn*4401* is 10 kb in size, is delimited by two 39-bp imperfect inverted repeat sequences, and is usually associated with 5-bp TSDs at both ends as a result of integration (48). The *bla*_KPC_-harboring Tn*4401* isoform and its TSD are important genetic markers that track with different plasmids, and consequently, they are useful plasmid genotyping tools (1). In this study, 43 Tn*4401b*, 2 Tn*4401a*, 24 Tn*4401d*, and 3 pKp048-like non-Tn*4401* mobile elements (NTM_KPC_) were identified (see Data Set S1). The two Tn*4401a* isoforms had the same TSD (ATTGA), which was initially described in plasmid pKpQIL (49). Interestingly, no apparent TSDs were identified upstream or downstream of Tn*4401d* in the 24 Tn*4401d*-bearing isolates. The 5-bp upstream (TCTCT) and downstream (GTTCT) adjacent sequences in the Tn*4401d*-bearing isolates are identical to those in IncFIA plasmids pBK30683 and pBK30661 (27). In contrast, nine different pairs of TSDs were found in the Tn*4401b*-bearing isolates (see Data Set S1). These data revealed the diverse Tn*4401* integration sites on different *Enterobacter* isolates.

**FIG 6:**
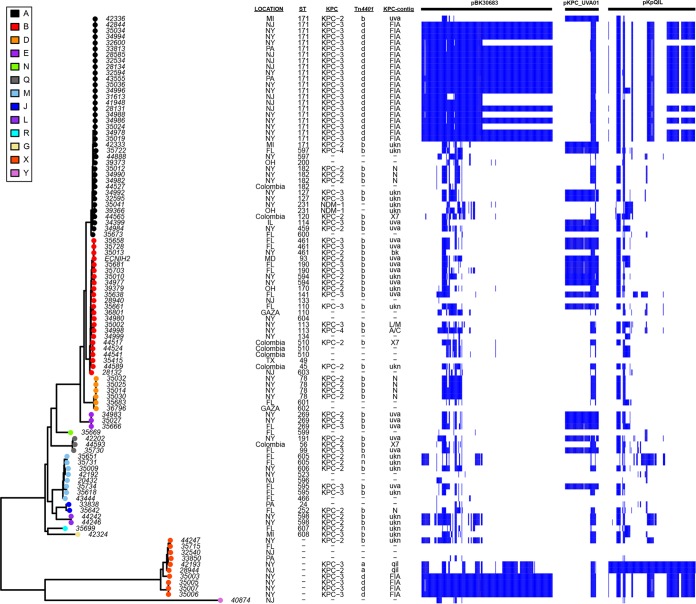
Representation of major KPC-harboring plasmids among 97 *Enterobacter* isolates. (Left) Core SNP phylogenetic tree generated by RAxML. Core SNPs were identified by kSNP v 3.0 (see Materials and Methods). (Middle) The metadata, including isolation location, ST, KPC variants, *Tn*4401 isoforms, and predicated *bla*_KPC_-harboring plasmids. (Right) Plasmid composition is illustrated by showing the BLASTn matches to each *Enterobacter* genome across all of the genes on the three reference plasmids, pBK30683, pKPC_UVA01, and pKpQIL. The blue bar denotes a minimal 95% nucleotide sequence identity to the plasmid genes. Abbreviations: uva, pKPC_UVA01-like plasmid; FIA, pBK30683 or pBK30661-like plasmid; Bk., pBK28610-like plasmid; qil, pKpQIL-like plasmid; ukn, *bla*_KPC_-harboring contigs could not be assigned to a known or novel plasmid group; n, pKp048-like non-*Tn*4401 mobile element (NTM_KPC_). Blue bars denote a ≥95% nucleotide sequence match to the plasmid genes.

Plasmid classification analysis (see Materials and Methods) successfully assigned the *bla*_KPC_-harboring contigs from 55 out of 72 isolates into a known or novel plasmid group (see Data Set S1) (Fig. 6). Among them, *bla*_KPC_ was found on an IncFIA plasmid (pBK30683- or pBK30661-like) in 24 isolates (24/55), and they all carry *bla*_KPC-3_ on the Tn*4401d* elements, flanked by 5-bp nonmatched adjacent sequences described above (Fig. 6). In addition, *bla*_KPC_ in 15 isolates was located on Tn*4401b* on the pKPC_UVA01-like plasmids with identical Tn*4401b* isoforms and TSDs. The *bla*_KPC_ genes in the remaining 16 isolates were associated with IncN-like (e.g., pKPC_FCF/3SP-like) (50) (*n* = 8), pKpQIL-like (*n* = 2), IncL/M-like (*n* = 1), IncA/C (p34998-239kb, *n* = 1), and pBK28610-like (*n* = 1) plasmids and a novel IncX (X7) plasmid (*n* = 3) (Fig. 6). Because of the short length of the *de novo* assembled contigs, we could not link 17 *bla*_KPC_-harboring and the 2 *bla*_NDM_-harboring contigs with any plasmid group.

We also examined the distribution of these *bla*_KPC_-harboring plasmids regarding their different phylogenetic groups (Fig. 6). The pBK30683-like plasmids were found in either group A or *E. aerogenes*. In group A, pBK30683-like plasmids were associated exclusively with ST171 isolates, suggesting the clonal spread of carbapenem resistance. In contrast, pKPC_UVA01-like conjugative plasmids appeared to be more promiscuous and have been found in isolates of various groups (A, B, E, M, and Q). Similarly, the newly identified *bla*_KPC_-harboring IncX7 plasmids were found in three isolates from three different phylogenetic groups (A, B, and Q). The spread of the same plasmid (e.g., pKPC_UVA01 and X7 plasmids) in unrelated strains suggested that the horizontal plasmid transfer contributed significantly to the dissemination of carbapenem resistance, which in part explains the aforementioned finding that plasmid gene content influences the topology of the gene content tree. Interestingly, pKpQIL-like plasmids, which are among the most common *bla*_KPC_-harboring plasmids in multiple *K. pneumoniae* STs and in other species (e.g., *Escherichia coli*) (21, 47, 49, 51–55), were identified in only two *E. aerogenes* isolates in this study, suggesting the likelihood of plasmid restriction between species or subspecies. One of them, plasmid pKpQIL-Ea (113,639 bp in length, from strain 28944), was completely sequenced in a previous study, and the sequence matches the prototype pKpQIL plasmid sequence from Israel, which is 113,637 bp in length (47).

### Molecular dissection of carbapenem-resistant *E. xiangfangensis* ST171.

**FIG 7:**
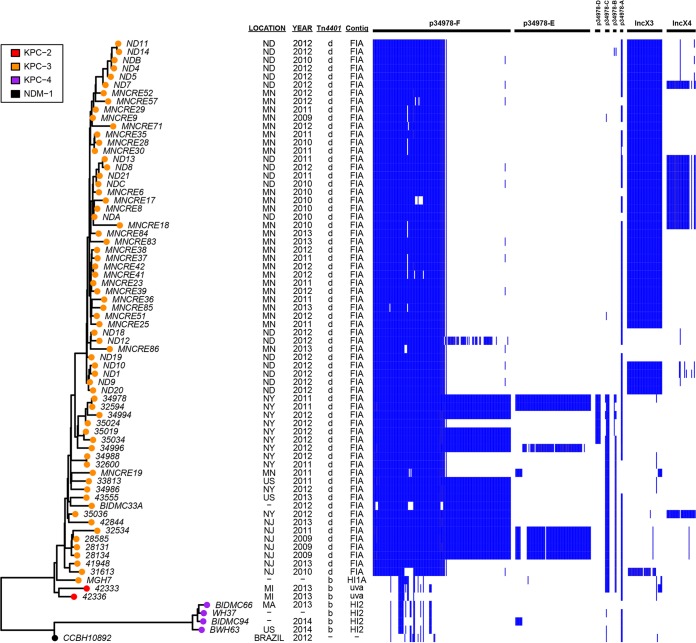
Representation of plasmid distribution among ST171 *Enterobacter* isolates. (Left) Core SNP phylogenetic tree generated by RAxML (see Materials and Methods). (Middle) The metadata, including isolation location, isolation year, *Tn*4401 isoforms, and predicated *bla*_KPC_-harboring plasmids. (Right) Plasmid composition is illustrated by showing the BLASTn matches to each *Enterobacter* genome across all of the genes on the reference plasmids. Six plasmids from completely sequenced *E. xiangfangensis* ST171 strain 34978, along with IncX3 plasmid pIncX-SHV (JN247852) and IncX4 plasmid pMNCRE44_4 (CP010880), were used as references. Blue bars denote a ≥95% nucleotide sequence match to the plasmid genes.

Currently, the dissemination of KPC in *K. pneumoniae* has been largely associated with a predominant clone, ST258, and closely related strains (1). It appears that in *Enterobacter* spp., *E. xiangfangensis* ST171 strains are an emerging clone that has been increasingly reported among CPE isolates from different hospitals in the United States (15, 56–58). In this study, ST171 is the most common ST among the 97 sequenced genomes, accounting for 22.7% of all genomes. Mining the *Enterobacter* spp. genome data from the present study and GenBank identified a total of 73 ST171 genomes, collected between 2009 and 2014 in New York, New Jersey, Massachusetts, Minnesota, North Dakota, and Brazil (Fig. 7). Considering all of the genomes analyzed, nearly 100% of the ST171 strains are positive for the *bla*_KPC_ gene (72/73), whereas only 28% of the non-ST171 strains carry the gene (71/257).

Core SNP analysis separated ST171 strains into two distinct clusters, with an average of ~373 core SNP differences and with a phylogeographic profile. The major cluster (cluster I) includes 68 genomes that differ from one another by an average of 42 SNPs. On the basis of their geographic distribution, cluster I can be divided into three subclades: ND/MN, NY/NJ, and MI. Previous genetic studies have indicated that the ND/MN subclade was clonally spreading in the North Dakota-Minnesota region (57). The only outlier isolate, MNCRE19, appears to be a member of the NY/NJ subclade, and it is possible that this isolate originated in the New York-New Jersey area.

Plasmid examination showed that isolates in the ND/MN and NY/NJ subclades all carry *bla*_KPC-3_ in a Tn*4401d* element. Further analysis demonstrated that the *bla*_KPC-3_ genes are located on the pBK30683-like IncFIA plasmid. As described above, pBK30683 is a cointegrate plasmid that has an ~70-kb pBK30661 IncFIA backbone and the addition of another *tra* operon-containing IncF plasmid region (27). Interestingly, the *bla*_KPC-3_-harboring IncFIA plasmids from the ND/MN subclade and five from NY/NJ subclade were only half the size of pBK30683, which is similar to pBK30661, and the *tra* operon-bearing IncFII plasmid region was not present (Fig. 7) (57).

Although the *bla*_KPC_-harboring plasmids are similar in ND/MN and NY/NJ isolates, their companion plasmids demonstrated a diverse distribution. NY/NJ isolates contain an additional p34978-70kb-like IncL/M plasmid, as well as ColE plasmids p34978-D and p34978-C, that is not present in ND/MN isolates. In contrast, most ND/MN isolates harbor the *bla*_SHV-12_-bearing IncX3 plasmids. Moreover, most isolates from North Dakota carry an additional IncX4 group plasmid, in comparison to Minnesota isolates (Fig. 7).

Cluster II contains four isolates harboring *bla*_KPC-4_ on a Tn*4401b* element on an IncHI2 plasmid that differ from one another by ~26 core SNPs. The outlier strain CCBH10892, harboring *bla*_NDM-1_ and isolated from Brazil, is divergent from clusters I and II and has 345 and 350 SNPs, in comparison to isolates from clades I and II, respectively.

## DISCUSSION

Identification of species and subspecies within the *E. cloacae* complex by phenotypic methods is challenging and often presents problems of reproducibility (59). Moreover, the genus *Enterobacter* is polyphyletic in nature and using 16S rRNA gene sequencing to classify strains into different species is challenging (60). There have been multiple proposals for transfers of species to and from this genus. As an example, *E. liquefaciens*, a member of the genus *Enterobacter*, was transferred to the genus *Serratia* in 1971 on the basis of DNA relatedness studies (60, 61).

We used genome sequences of many diverse *Enterobacter* species isolates to refine species designations for this genus. Using ANI and SNP analyses, we placed 447 *Enterobacter* genomes into 19 phylogenomic groups—18 groups (A to R) in the *E. cloacae* complex and 1 in *E. aerogenes*. Groups containing type strains were given specific species names; otherwise, just “*cloacae* complex” was used with the strain name. The distinct phylogenomic grouping of *E. aerogenes* on the basis of whole-genome sequencing clearly supports the previously proposed argument to rename this species *K. aerogenes* (40).

Carbapenemase-producing *Enterobacter* spp. are increasingly being identified in clinical settings; however, knowledge regarding their genomic nature, population structure, and resistance plasmid characteristics remains limited. Carbapenemase-producing strains were found in nearly every group (Fig. 2). The genomic signatures (including resistance determinants, Tn*4401* variants, plasmids, and host genomes) strongly suggested that both horizontal gene transfer and clonal expansion have contributed to the dissemination of carbapenem-resistant strains in this genus. We also showed that plasmid gene content can influence the topology of trees drawn from the presence/absence of gene content and therefore should be removed prior to the generation of gene content trees from pangenome studies.

Our study demonstrated that the spread of *bla*_KPC_ among *Enterobacter* spp. is due to multiple complex mechanisms. First, we have observed the acquisition of a *bla*_KPC_-harboring plasmid by a specific host strain, followed by clonal expansion into different geographic regions. The example is the clonal spread of *E. xiangfangensis* ST171 harboring *bla*_KPC-3_ on an IncFIA plasmid (Fig. 7). IncFIA plasmid pBK30683-like was one of the predominant *bla*_KPC_-harboring plasmids in *K. pneumoniae* and has been circulating in New York-New Jersey hospitals since the early 2000s (27). Subsequently, this plasmid was identified in *Enterobacter* spp. in 2009 from different hospitals in the New York-New Jersey area (27). We suspected that the pBK30683-like plasmids in *Enterobacter* spp. may originate through horizontal plasmid transfer from *K. pneumoniae*. Of note, pBK30683-like plasmids have two different forms, the nonconjugative pBK30661 type and the conjugative/cointegrate pBK30683 type (27). Interestingly, the MN/ND ST171 clade all carry the pBK30661-type plasmid, and in contrast, the majority of NY/NJ isolates (except for 35024, 34988, 32600, 41948, and 31613) harbored the cointegrate pBK30683-type plasmid (Fig. 7). It is hypothesized that the carbapenem-resistant ST171 MN/ND isolates were descendants of an ST171 strain harboring a nonconjugative IncFIA plasmid or originated by the acquisition of an IncFIA cointegrate plasmid by a local ST171 strain, and the IncF region on pBK30683 was subsequently lost. The nonconjugative IncFIA plasmid in MN/ND ST171 strains is not able to conjugate and can only be spread clonally, until it forms novel cointegrates as reported elsewhere (21, 57, 62).

Second, horizontal transfer of a common plasmid across different phylogenomic clades has occurred in *Enterobacter* spp. One example is the identification of pKPC_UVA01-like plasmids in multiple phylogenomic groups (Fig. 6). pKPC_UVA01, initially described in *K. pneumoniae* in a Virginia hospital system, has now been frequently found in various species, including *K. pneumoniae*, *E. cloacae*, *E. aerogenes*, *E. coli*, *K. oxytoca*, and *Citrobacter freundii* (25, 63). A recent study indicated that in pKPC_UVA01, Tn*4401b* was integrated into *bla*_TEM-1_-containing transposon Tn*2* and the Tn*2*-Tn*4401* nested transposon was translocated into different plasmid backbones (63). The authors of that study warned that the use of reference-based plasmid interpretation may produce misleading results. In this study, only the *de novo* assembled contigs containing both Tn*4401* and additional pKPC_UVA01 core plasmid genes (e.g., replicons) were considered in the analysis (see Materials and Methods). Using this approach, 15 pKPC_UVA01-like plasmids were found. Our results showed that the pKPC_UVA01-like plasmids spread into different groups of *Enterobacter* spp. and were identified in different geographic regions (New York, Michigan, Maryland, Illinois, and Florida). In addition, six pKPC_UVA01-like plasmids carry *bla*_KPC-2_, while nine harbor *bla*_KPC-3_, indicating multiple independent acquisitions of different *bla*_KPC_ variants with Tn*4401* integrated into a hot spot on the same plasmid backbone. Another example is the IncX7 plasmid harboring *bla*_KPC-2_ from Columbia. We identified nearly identical ~47-kb contigs containing Tn*4401b* and IncX7 plasmid core operons (encoding replication, stability, and transfer) in 44565 (group A), 44517 (group B), and 44593 (group Q).

Third, multiple discrete acquisition events involving *bla*_KPC_-containing elements (e.g., Tn*4401*) have contributed to the dissemination of carbapenem resistance in *Enterobacter* spp. We found that Tn*4401d* and Tn*4401a* in the 72 *bla*_KPC_-bearing isolates were associated with pBK30683- and pKpQIL-like plasmids, respectively. In contrast, Tn*4401b* showed a high degree of diversity regarding its TSDs and harboring plasmids. Nine unique TSDs were found to be associated with Tn*4401b*, suggesting the repeated acquisition of Tn*4401* on different plasmids or different locations on the same plasmid (e.g., IncN) backbones (see Data Set S1).

Finally, there are common *bla*_KPC_-harboring plasmids in *K. pneumoniae* that are nearly absent in *Enterobacter* spp. IncF_IIK_ pKpQIL-like plasmids are among the most predominant *bla*_KPC_-harboring plasmids in *K. pneumoniae* and *E. coli* from the northwestern United States and other geographic regions (21, 47, 49, 51–55), but interestingly, among the 72 *bla*_KPC_-harboring isolates, only 2 *E. aerogenes* isolates have been found to carry pKpQIL. Of note, the time period of the present study overlaps our previous molecular surveillance study that investigated the prevalence of pKpQIL in *K. pneumoniae* (47). Our previous study found the pKpQIL-like plasmids accounted for as much as 35% of the *bla*_KPC_-harboring plasmids in *K. pneumoniae* (47). In contrast, in the present study, only 3% (2/72) of the *bla*_KPC_-harboring plasmids are pKpQIL. In addition, searches of the public *Enterobacter* genomes did not identify any additional pKpQIL replicons (positive for both IncFII_K_ and IncFIBqil). This finding may mean that IncF_IIK_ pKpQIL plasmids are not “compatible” or “stable” in the *Enterobacter* host.

*E. xiangfangensis* ST171 strains are the most predominant *Enterobacter* clones reported in our study and in the public databases. However, unlike the epidemic *K. pneumoniae* ST258 strains, where two distinct subgroups arose because of recombination in the K antigen-encoding capsule polysaccharide biosynthesis gene (*cps*) region (64), the ST171 isolates likely evolved from a common ancestor and two major clusters each acquired distinct resistance plasmids. Several outbreaks and clonal spread have been linked to the ST171 isolates, suggesting that this genetic background contributes to the epidemiological success of ST171.

Our study has limitations. More than half of the isolates in this study are from the New York-New Jersey area, and consequently, this capture likely does not define the overall molecular epidemiology of carbapenem-resistant *Enterobacter* spp. in the United States or worldwide. In addition, as KPC is the main carbapenemase in the United States and most of the isolates harbored *bla*_KPC_, our study was not able to examine the genomic structures of carbapenem-resistant *Enterobacter* strains producing other carbapenemases, such as NDM, OXA-48, VIM, and IMP. A further study including *Enterobacter* isolates producing other carbapenemases will likely reveal additional genomic characteristics contributing to carbapenem resistance. Moreover, only six isolates were characterized by long-read sequencing, which produced closed genomes, and the results of most isolates were interpreted on the basis of *de novo* assemblies of short-read data. Because of the limitation of short-read data, the genetic structures, particularly repetitive regions, are hard to resolve. This is largely the reason why we were not able to define the structures of *bla*_NDM_-harboring plasmids and why no conclusive *bla*_KPC_ plasmids were identified in 19 out of 72 KPC-producing isolates. Overall, however, our deep genomic analysis across the *Enterobacteriaceae* family has clearly defined the phylogeny and revealed distinct genomic signatures linked to carbapenem resistance.

The data presented here represent the first comprehensive study of phylogenomic relationships, antibiotic resistance, and plasmid discrimination of carbapenem-resistant *Enterobacter* spp. Our study suggests that acquisition of specific plasmids, successful host clones, and plasmid-host combinations are driving the molecular evolution of carbapenem resistance in the *Enterobacter* genus. Carbapenem resistance due to *bla*_KPC_ has resulted in a pathogen that is difficult to treat, and in many instances, the clinical options are limited to less effective therapy. Improved understanding of the relationships among *Enterobacter* species and strains and the genetic context of resistance genes that they carry will be of significant value in tracking these organisms in a clinical context and in developing strategies to limit their spread.

## MATERIALS AND METHODS

### Bacterial isolates.

Ninety-seven *Enterobacter* sp. clinical isolates were collected from patients at 16 health care institutions in the United States (New York City [*n* = 43], New Jersey [*n* = 13], Florida [*n* = 20], Illinois [*n* = 1], Michigan [*n* = 3], Ohio [*n* = 3], and Pennsylvania [*n* = 4], Texas [*n* = 1]), South America (Colombia [*n* = 7]), and the Mediterranean region (Gaza [*n* = 2]). We selected six representative KPC-harboring *Enterobacter* isolates for complete genome sequencing on the basis of the presence of *bla*_KPC_ gene variants, Tn*4401* isoforms, multilocus sequence typing, and geographic and temporal distribution. *Enterobacter* species isolates were cultured overnight in lysogeny broth at 37°C for subsequent isolation of DNA for genome sequencing (see below). MICs were determined by broth microdilution in cation-adjusted Mueller-Hinton broth according to Clinical and Laboratory Standards Institute methods with Sensititre GNX2F panels (Thermo Fisher Scientific).

### *Enterobacter* sp. *de novo* DNA sequencing.

*Enterobacter* sp. DNA was isolated from overnight cultures with a MasterPure Gram Positive DNA Purification kit (Epicentre, United States) as recommended by the manufacturer. Libraries were prepared for sequencing with Illumina NexteraXT kits and sequenced on an Illumina NextSeq with paired 150-base sequence reads. In general, >100-fold coverage was obtained for each genome. Each read set was assembled individually with *SPAdes* (37) and annotated with NCBI’s PGAAP pipeline (http://www.ncbi.nlm.nih.gov/genome/annotation_prok/). PacBio libraries were constructed and sequenced according to the manufacturer’s recommendations to ~100× coverage.

### Characterization of *Enterobacter* species strains.

*In silico* multilocus sequence typing of 390 *E. cloacae* complex strains was performed with the MLST 1.8 online server (24, 38). The antimicrobial resistance genes and plasmid replicons in the sequenced *Enterobacter* isolates were identified by BLAST searching with the databases of ResFinder 2.1 (39) and PlasmidFinder 1.3 (65). Additional novel plasmid replicons from the present study were included in the analysis as well.

### kSNP *Enterobacter* trees.

A phylogenetic tree was inferred from SNPs identified by kSNP v 3.0 (30) by using a k-mer length of 19 nucleotides and a requirement that at least 80% of the genomes (i.e., 303 genomes) have a nucleotide at a given SNP position in order for the SNP to be considered to be core and included in tree building. A total of 501,576 core SNP positions were identified. These SNPs were used to infer a maximum-likelihood tree with RAxML (31) with 100 bootstrap replicates. The resulting tree was rendered with metadata annotated with GraPhlAn (66).

### Universal marker tree.

A total of 248 publically available genomes belonging to 48 *Enterobacteriaceae* genera were downloaded from GenBank. At least five genomes from each genus were included, prioritizing type strains, closed genomes, high-quality whole-genome sequences (e.g., contig N50 of 20 kbp or greater and ≤500 contigs), and phylogenetic diversity. In cases where there were fewer than five genome representatives of a given genus, all of the genomes meeting minimum assembly quality thresholds were downloaded. Twenty-six different universal marker genes (*atpD*, *rplA*, *rplB*, *rplC*, *rplE*, *rplF*, *rplK*, *rplM*, *rplN*, *rplP*, *rplR*, *rplV*, *rpoA*, *rpsB*, *rpsC*, *rpsD*, *rpsE*, *rpsG*, *rpsH*, *rpsI*, *rpsK*, *rpsL*, *rpsM*, *rpsO*, *rpsQ*, and *secY*) were used as seed sequences to identify the corresponding gene locus in each genome. These genes are universally conserved among bacteria and produce monophyletic phylogenies, suggesting that they undergo minimal horizontal transfer (67–69). Individual genes were identified by the J. Craig Venter Institute gene locus-typing program LOCUST (unpublished data), concatenated, and aligned with MUSCLE (70). The resulting alignment was used to generate a maximum-likelihood tree from 100 bootstrapped replicates with RAxML (31). The resulting tree was rendered with metadata annotated with GraPhlAn (66).

### Pangenome analysis.

Clusters of orthologous proteins were generated with version 3.24 of PanOCT (41) as previously described (71, 72). Plots and calculations of pangenome size, new genes discovered, and pangenome status (open versus closed) were also determined as described previously (71).

### ANI.

ANI of *Enterobacter* genomes was performed by PanOCT version 3.24, which has been modified to also accept nucleotide sequences as an alternative input to amino acid sequences as in previous versions.

### Plasmid classification.

The *bla*_KPC_-harboring contigs were extracted from the *de novo* assemblies, followed by a BLASTn search against publicly available plasmid sequences in GenBank. If the contig coexisted with other plasmid core elements (e.g., the replicon) and showed >99% identity to and >90% query coverage of a known plasmid, the contig was preliminarily classified as the reference-like plasmid (e.g., pUAV01_KPC-like). The contig should have the same Tn*4401* variant and TSD as the reference plasmid. The contigs were further aligned to the putative references and visually inspected to confirm the plasmid contents with Geneious (version 8.1; Biomatters Ltd., Auckland, New Zealand). Furthermore, BLASTn comparisons of each isolate’s *de novo* assembly and the reference plasmid were conducted, and the presence of a reference-like plasmid was defined as ≥99% sequence identity over ≥80% of the length of the reference. The plasmid content diagrams were generated by R with the plotTree.R script (available at https://github.com/katholt/RedDog).

### Variant detection.

Single-nucleotide variant (SNV) analysis for ST171 isolates was performed with the RedDog pipeline (https://github.com/katholt/RedDog). In brief, the Illumina reads were mapped to ST171 reference strain 34978 with Bowtie2 (73), and SNVs were called by SAMtools (74) (Phred score, ≥30; read depth, ≥10). Consensus alleles at all SNV sites were then extracted with SAMtools (74). SNV sites present in all ST171 genomes were concatenated to generate a core SNV alignment for phylogenetic analysis. Draft genomes from the public database were each shredded into 1 million 100-bp reads (with SAMtools wgsim) and subjected to the same analysis as the Illumina reads.

### Accession number(s).

All of the genomes determined in this study are available at NCBI under BioProject no. PRJNA259658.

## SUPPLEMENTAL MATERIAL

Data Set S1  Comprehensive table with details about the 97 clinical isolates and six type strains sequenced in this study. Download Data Set S1, XLSX file, 0.04 MB

Data Set S2  Details of 351 *Enterobacter* genomes from GenBank. Download Data Set S2, XLSX file, 0.04 MB

Text S1  Phylogenetic structure, subspecies identification, and pangenome analysis of *Enterobacter* spp. Download Text S1, DOCX file, 0.03 MB

Figure S1  Comparison of *bla*_KPC_-harboring plasmids from four PacBio sequenced strains. Light blue shading denotes shared regions of homology with 99% identities. Light gray shading denotes homologous regions acquired from another plasmid (pENT-c88). Open reading frames are represented by arrows colored on the basis of the predicted gene function (see key, top right). Plasmid scaffold regions are represented by orange arrows. The genes associated with the *tra* locus are represented by green arrows, and the antimicrobial resistance genes are represented by red arrows. Replication-associated genes are represented by dark blue arrows, while the accessory genes are represented by yellow arrows. Download Figure S1, PDF file, 0.3 MB

Figure S2  Comparison of *bla*_KPC-4_-harboring plasmids from BK34998. Light blue shading denotes IncA/C plasmid backbone regions shared among three plasmids, pRA1, p34998-E, and p35734-C. Light gray shading denotes region of homology surrounding *bla*_KPC-4_ from IncN plasmid pBK31551. Open reading frames are represented by arrows colored on the basis of the predicted gene function (see key, top right). Download Figure S2, PDF file, 0.3 MB

Figure S3  Plasmid gene content can influence gene content tree topology. An unrooted neighbor-joining tree was constructed by Neighbor by using the PanOCT Jaccard pairwise distance matrix of orthologous gene clusters with (A) and without (B) plasmid genes. Coloring is by assigned *E. cloacae* complex groupings A to E (see key and Fig. 5). Nodes are labeled by strain name. The asterisks mark those branches of the tree with mixed group assignments prior to the removal of clusters containing known plasmid genes from the complete genomes used in the pangenome analysis. The scale bars indicate distance. Download Figure S3, PDF file, 0.8 MB

Figure S4  Phylogenetic SNP tree of *Enterobacter* genomes. A whole-genome core SNP tree was constructed for 447 *Enterobacter* sp. genomes by using kSNP (30) and RAxML (31) (see Materials and Methods). The dendrogram was generated with FigTree v 1.4.2 (http://tree.bio.ed.ac.uk/software/figtree/). This data set included genomes within the 379 *E. cloacae* complex (black) and 68 *E. aerogenes* (blue) genomes used in this study. The scale bar indicates the number of nucleotide substitutions. Download Figure S4, PDF file, 0.6 MB

Table S1  Resistance genes and incompatibility groups of plasmids from six PacBio sequenced *Enterobacter* strains. Table S1, DOCX file, 0.01 MB
