# Supplementary material for: Comprehensive Genome Analysis of Carbapenemase-Producing Enterobacter spp.: New Insights into Phylogeny, Population Structure, and Resistance Mechanisms
Source: mBio. 2016 Dec 13;7(6):e02093-16. doi: 10.1128/mBio.02093-16 (PMC5156309; doi:10.1128/mBio.02093-16)
Supplement: Text S1 — Phylogenetic structure, subspecies identification, and pangenome analysis of Enterobacter spp. Download [file mbo006163111s1.docx]

***Phylogenetic structure of Enterobacter species***

Historically, bacterial species definition has been based on 70% DNA-DNA hybridization (DDH) and 16S rRNA gene sequences; however, more recently these methods have been replaced by *in silico* methods such as average nucleotide identify (ANI) (1) and a genome-to-genome distance (GGD) calculation (2). The recommended ANI threshold to define species groups was previously set to 94% to approximate the 70% DDH cut-off, but was adjusted to 95-96% ANI (3). More recently, it has been shown that there is a sharp decline in the number of genomes with an ANI < 96.5% for genomes in GenBank with the same species name (4). ANI and SNP phylogeny were concordant in clustering the genomes into phylogenetic groups.

***Identifying members of the Enterobacter cloacae complex to the subspecies level***

In a seminal work, Hoffmann and Roggenkamp (5) investigated the genetic structure of the *E. cloacae* complex by a combination of sequencing of the three housekeeping genes *hsp60*, *rpoB*, and *hemB;* and PCR-restriction fragment length polymorphism (PCR-RFLP) analysis of *ampC*. They defined 12 genetic clusters (I-XII) based most exhaustively on the *hsp60* sequencing. Three of the clusters (cluster III, 58 strains; cluster VI, 28 strains; and cluster VIII, 59 strains) accounted for 70% of the 206 strains studied. The authors noted that “Only 3% of our study strains clustered with the type strain of *E. cloacae*.” (cluster XI), “We found that 3% of our study strains clustered around the *E. hormaechei* type strain.” (cluster VII), and “Our clusters VI and VIII were closely related to *E. hormaechei* cluster VII. DNA-DNA hybridization studies are needed to verify whether these clusters form a common DNA relatedness group allowing emending and broadening of the species description of *E. hormaechei*.”

Hoffmann *et al*. (6) followed up with a characterization of clusters VI, VII, and VIII asserting based on DNA hybridization that these clusters were subspecies of the same species and since cluster VII contained the type strain for *E. hormaechei* they named cluster VII *E. hormaechei* subsp. *hormaechei*, cluster VI *E. hormaechei* subsp. *oharae*, and cluster VIII *E. hormaechei* subsp. *steigerwaltii*. Forty-eight strains were characterized using 129 biochemical tests showing that there were phenotypic differences between the subspecies. However, the authors did not include the other predominant cluster (III) in their analysis, and these subspecies names remain to be validly published.

Gu *et al*. (7) defined *Enterobacter xiangfangensis* using a phylogenetic tree based upon concatenated partial *rpoB*, *atpD*, *gyrB* and *infB* gene sequences from a novel isolate and existing type strains where *E. xiangfangensis* was closest to *E. hormaechei* in the tree.

**Pan-genome of *E. hormaechei***: *Core pan-genome.*

To explore the potential functional significance of distinct *E. hormaechei* subspecies, the pan-genome of all available *E. hormaechei* genomes was determined using the PanOCT software suite (8, 9)**.** PanOCT uses genome co-location information to assist in inference of orthologous gene relationships in large sets of closely related genomes. The resulting sets of genes that are identified in all genomes (“core” genes) and genes that are shared by subsets of genomes can be used to infer common and variable functional traits among groups of strains.

Regarding pan-genome size, we followed the convention of merging clusters of paralogous proteins (9), which resulted in a reduction in the number of clusters from 21,213 to 18,223. To predict the maximum pan-genome size (i.e., the total number of genes, including core/universal, novel/unique/strain-specific and periphery/dispensable genes) a pan-genome model was implemented as described previously (9, 10) (**Fig 4b**). The maximum pan-genome size was extrapolated and determined to be 19,337 ± 115 genes. To determine whether the *E. hormaechei* pan-genome is open or closed, the number of new genes identified (i.e., unique or strain-specific genes) for each genome added was determined as described previously (9, 10) (**Fig 4b**). A pan-genome is considered closed when sequencing the genomes of additional isolates fails to expand the pan-genome (i.e., the entire gene repertoire has been discovered). The exponent (α) in the power law function (n = κN^-α^) indicates whether the pan-genome is open (α ≤ 1) or closed (α > 1) (11). Using this equation, the pan-genome of *E. hormaechei* appeared to be open (α = 0.8182 ± 0.004; **Fig 4b**). The number of new genes found for each genome added to the pan-genome was determined from the exponential decay function to be 26 ± 1.6 (**Fig 4c**).

**References**

1. **Konstantinidis KT, Tiedje JM.** 2005. Genomic insights that advance the species definition for prokaryotes. Proc Natl Acad Sci U S A **102:**2567-2572.

2. **Meier-Kolthoff JP, Auch AF, Klenk HP, Goker M.** 2013. Genome sequence-based species delimitation with confidence intervals and improved distance functions. BMC Bioinformatics **14:**60.

3. **Richter M, Rossello-Mora R.** 2009. Shifting the genomic gold standard for the prokaryotic species definition. Proc Natl Acad Sci U S A **106:**19126-19131.

4. **Varghese NJ, Mukherjee S, Ivanova N, Konstantinidis KT, Mavrommatis K, Kyrpides NC, Pati A.** 2015. Microbial species delineation using whole genome sequences. Nucleic Acids Res **43:**6761-6771.

5. **Hoffmann H, Roggenkamp A.** 2003. Population genetics of the nomenspecies Enterobacter cloacae. Appl Environ Microbiol **69:**5306-5318.

6. **Hoffmann H, Stindl S, Ludwig W, Stumpf A, Mehlen A, Heesemann J, Monget D, Schleifer KH, Roggenkamp A.** 2005. Reassignment of enterobacter dissolvens to Enterobacter cloacae as E. cloacae subspecies dissolvens comb. nov. and emended description of Enterobacter asburiae and Enterobacter kobei. Syst Appl Microbiol **28:**196-205.

7. **Gu CT, Li CY, Yang LJ, Huo GC.** 2014. Enterobacter xiangfangensis sp. nov., isolated from Chinese traditional sourdough, and reclassification of Enterobacter sacchari Zhu et al. 2013 as Kosakonia sacchari comb. nov. Int J Syst Evol Microbiol **64:**2650-2656.

8. **Fouts DE, Brinkac L, Beck E, Inman J, Sutton G.** 2012. PanOCT: Automated Clustering of Orthologs Using Conserved Gene Neighborhood for Pan-Genomic Analysis of Bacterial Strains and Closely Related Species. Nucleic Acids Res **40:**e172.

9. **Adler A, Hussein O, Ben-David D, Masarwa S, Navon-Venezia S, Schwaber MJ, Carmeli Y.** 2015. Persistence of *Klebsiella pneumoniae* ST258 as the predominant clone of carbapenemase-producing *Enterobacteriaceae* in post-acute-care hospitals in Israel, 2008-13. J Antimicrob Chemother **70:**89-92.

10. **Tettelin H, Masignani V, Cieslewicz MJ, Donati C, Medini D, Ward NL, Angiuoli SV, Crabtree J, Jones AL, Durkin AS, Deboy RT, Davidsen TM, Mora M, Scarselli M, Margarit y Ros I, Peterson JD, Hauser CR, Sundaram JP, Nelson WC, Madupu R, Brinkac LM, Dodson RJ, Rosovitz MJ, Sullivan SA, Daugherty SC, Haft DH, Selengut J, Gwinn ML, Zhou L, Zafar N, Khouri H, Radune D, Dimitrov G, Watkins K, O'Connor KJ, Smith S, Utterback TR, White O, Rubens CE, Grandi G, Madoff LC, Kasper DL, Telford JL, Wessels MR, Rappuoli R, Fraser CM.** 2005. Genome analysis of multiple pathogenic isolates of *Streptococcus agalactiae*: implications for the microbial "pan-genome". Proc Natl Acad Sci U S A **102:**13950-13955.

11. **Tettelin H, Riley D, Cattuto C, Medini D.** 2008. Comparative genomics: the bacterial pan-genome. Curr Opin Microbiol **11:**472-477.
