# Supplementary material for: Comprehensive Genome Analysis of Carbapenemase-Producing Enterobacter spp.: New Insights into Phylogeny, Population Structure, and Resistance Mechanisms
Source: mBio. 2016 Dec 13;7(6):e02093-16. doi: 10.1128/mBio.02093-16 (PMC5156309; doi:10.1128/mBio.02093-16)
Supplement: Figure S1 — Comparison of blaKPC-harboring plasmids from four PacBio sequenced strains. Light blue shading denotes shared regions of homology with 99% identities. Light gray shading denotes homologous regions acquired from another plasmid (pENT-c88). Open reading frames are represented by arrows colored on the basis of the predicted gene function (see key, top right). Plasmid scaffold regions are represented by orange arrows. The genes associated with the tra locus are represented by green arrows, and the antimicrobial resistance genes are represented by red arrows. Replication-associated genes are represented by dark blue arrows, while the accessory genes are represented by yellow arrows. Download [file mbo006163111sf1.pdf]

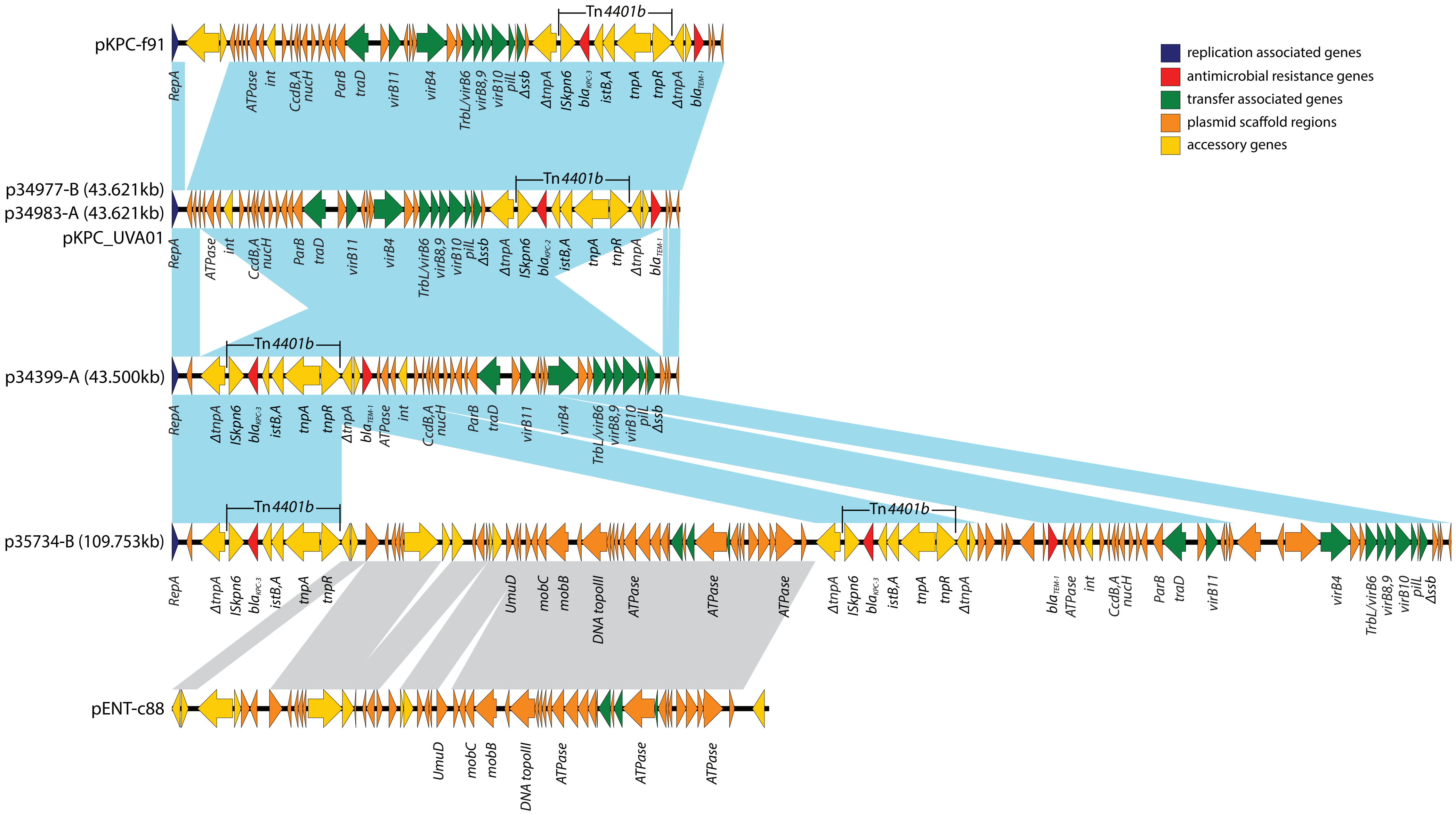

Figure S1: Comparison of *bla*$_{KPC}$ harboring plasmids from 4 PacBio sequenced strains.
The light-blue shading denotes shared regions of homology with 99% identities. Light-gray shading denotes homologous regions acquired from other plasmids (pENT-c88). Open reading frames (ORFs) are portrayed by arrows and colored based on the predicted gene function (see key, top right). Plasmid scaffold regions are indicated by orange arrows. The genes associated with the tra locus are indicated by green arrows, and the antimicrobial resistance genes are denoted as red arrows. Replication associated genes are indicated by dark-blue arrows, while the accessory genes are indicated by yellow arrows.
