# Supplementary material for: Comprehensive Genome Analysis of Carbapenemase-Producing Enterobacter spp.: New Insights into Phylogeny, Population Structure, and Resistance Mechanisms
Source: mBio. 2016 Dec 13;7(6):e02093-16. doi: 10.1128/mBio.02093-16 (PMC5156309; doi:10.1128/mBio.02093-16)
Supplement: Figure S2 — Comparison of blaKPC-4-harboring plasmids from BK34998. Light blue shading denotes IncA/C plasmid backbone regions shared among three plasmids, pRA1, p34998-E, and p35734-C. Light gray shading denotes region of homology surrounding blaKPC-4 from IncN plasmid pBK31551. Open reading frames are represented by arrows colored on the basis of the predicted gene function (see key, top right). Download [file mbo006163111sf2.pdf]

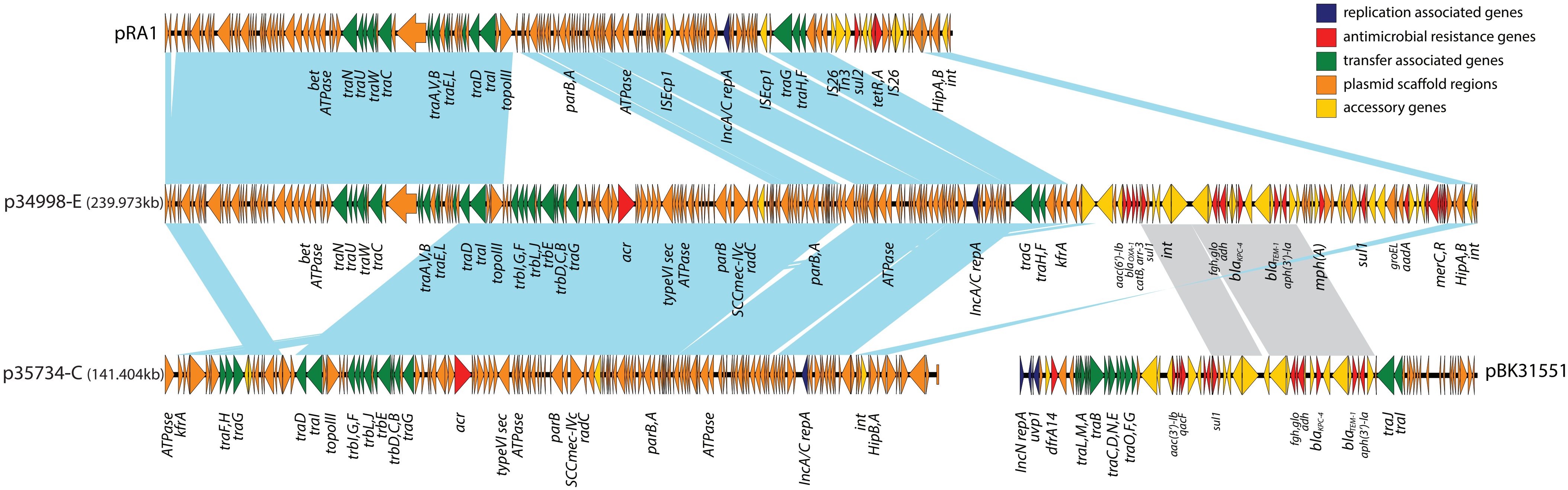


**Figure S2: Comparison of *bla*$_{KPC-4}$ harboring plasmid from BK34998.**
The light-blue shading denotes regions of IncA/C plasmid backbone shared among three plasmids, pRA1, p34998-E, and p35734-C. Light-gray shading denotes region of homology surrounding *bla*$_{KPC-4}$ from an IncN plasmid pBK31551. ORFs are symbolized by arrows and colored based on the predicted gene function (see key, top right).
