# Supplementary material for: Comprehensive Genome Analysis of Carbapenemase-Producing Enterobacter spp.: New Insights into Phylogeny, Population Structure, and Resistance Mechanisms
Source: mBio. 2016 Dec 13;7(6):e02093-16. doi: 10.1128/mBio.02093-16 (PMC5156309; doi:10.1128/mBio.02093-16)
Supplement: Figure S3 — Plasmid gene content can influence gene content tree topology. An unrooted neighbor-joining tree was constructed by Neighbor by using the PanOCT Jaccard pairwise distance matrix of orthologous gene clusters with (A) and without (B) plasmid genes. Coloring is by assigned E. cloacae complex groupings A to E (see key and Fig. 5). Nodes are labeled by strain name. The asterisks mark those branches of the tree with mixed group assignments prior to the removal of clusters containing known plasmid genes from the complete genomes used in the pangenome analysis. The scale bars indicate distance. Download [file mbo006163111sf3.pdf]

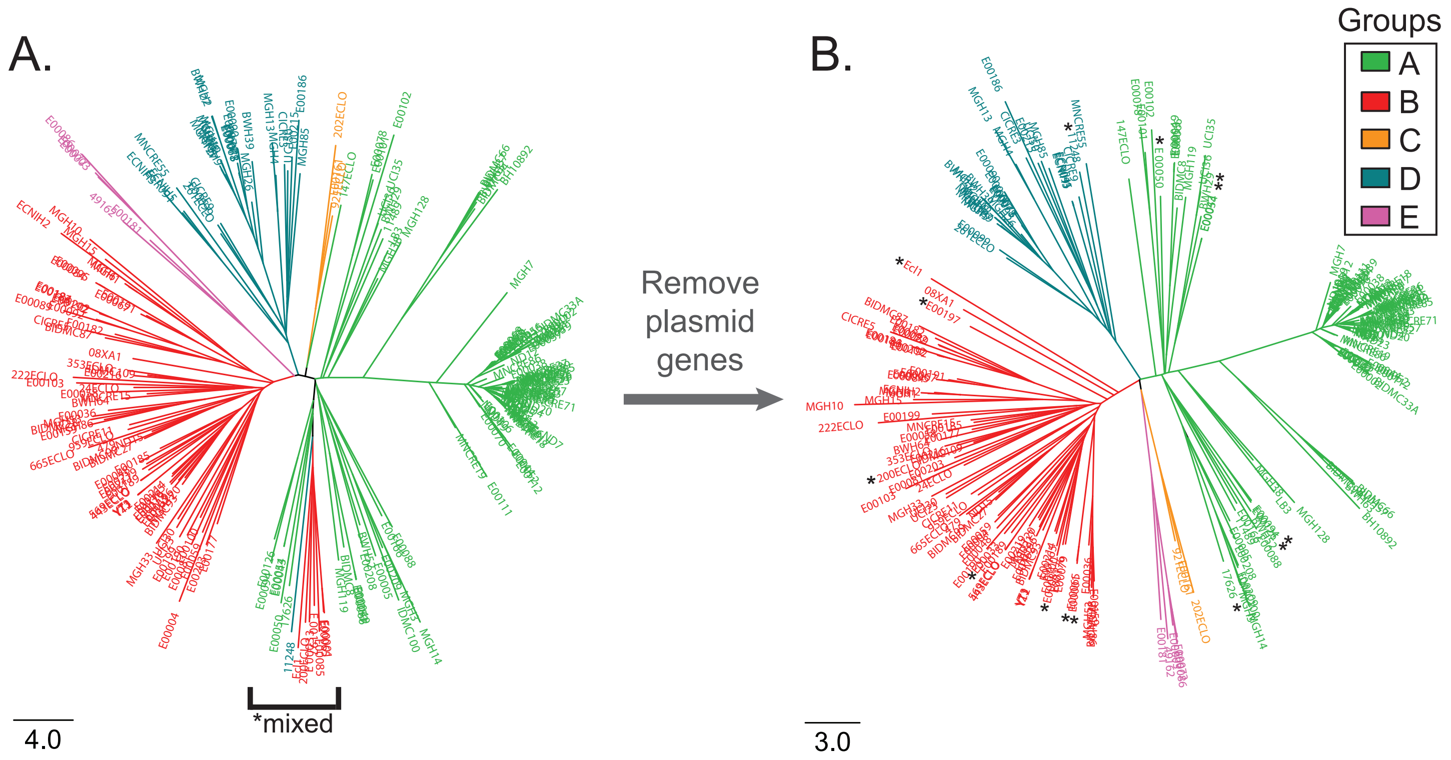

Figure S3: Plasmid gene content can influence gene content tree topology.
An unrooted Neighbor-Joining tree was constructed by Neighbor (83, 84) using the PanOCT Jaccard pairwise distance matrix of orthologous gene clusters with (A) and without plasmid genes (B). Coloring is by assigned *Enterobacter cloacae* complex groupings A-E (see key and Fig. 5). Nodes are labeled by strain name. The asterisk marks those branches of the tree with mixed group assignments prior to the removal of clusters containing known plasmid genes from the complete genomes used in the pan-genome analysis. The scale bar indicates distance.
