# Supplementary material for: Comprehensive Genome Analysis of Carbapenemase-Producing Enterobacter spp.: New Insights into Phylogeny, Population Structure, and Resistance Mechanisms
Source: mBio. 2016 Dec 13;7(6):e02093-16. doi: 10.1128/mBio.02093-16 (PMC5156309; doi:10.1128/mBio.02093-16)
Supplement: Figure S4 — Phylogenetic SNP tree of Enterobacter genomes. A whole-genome core SNP tree was constructed for 447 Enterobacter sp. genomes by using kSNP (30) and RAxML (31) (see Materials and Methods). The dendrogram was generated with FigTree v 1.4.2 (http://tree.bio.ed.ac.uk/software/figtree/). This data set included genomes within the 379 E. cloacae complex (black) and 68 E. aerogenes (blue) genomes used in this study. The scale bar indicates the number of nucleotide substitutions. Download [file mbo006163111sf4.pdf]

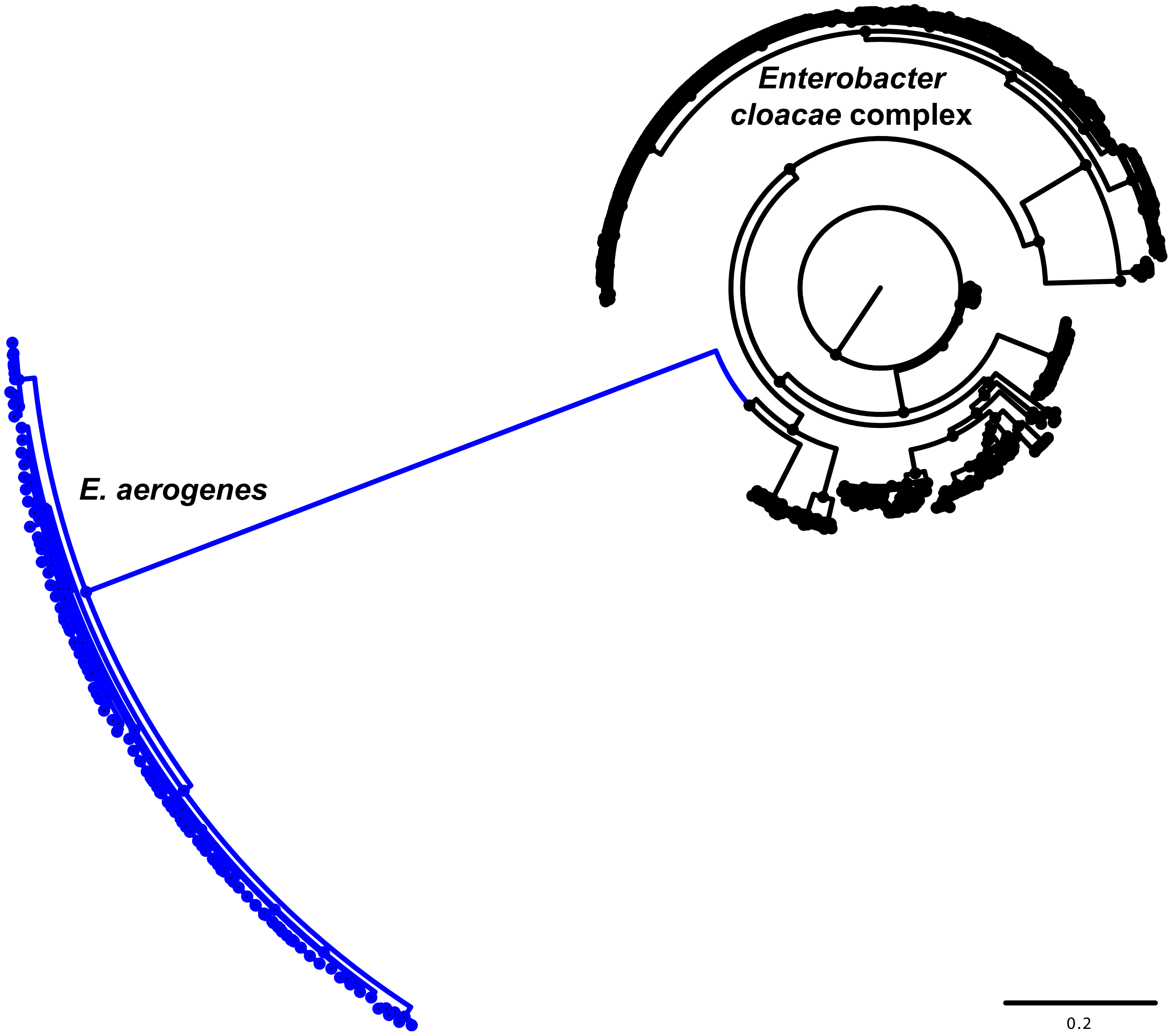

Figure S4. Phylogenetic SNP tree of *Enterobacter* genomes.
A whole genome core SNP tree was constructed for 447 *Enterobacter* spp. genomes using kSNP (30) and RaXML (31) (see Methods). The dendrogram was generated using FigTree v1.4.2 (http://tree.bio.ed.ac.uk/software/figtree/). This dataset included genomes within the 379 *E. cloacae* complex (black) and 68 *E. aerogenes* (blue). The scale bar indicates the number of nucleotide substitutions.
