# Supplementary material for: Comprehensive Genome Analysis of Carbapenemase-Producing Enterobacter spp.: New Insights into Phylogeny, Population Structure, and Resistance Mechanisms
Source: mBio. 2016 Dec 13;7(6):e02093-16. doi: 10.1128/mBio.02093-16 (PMC5156309; doi:10.1128/mBio.02093-16)
Supplement: Table S1 — Resistance genes and incompatibility groups of plasmids from six PacBio sequenced Enterobacter strains. [file mbo006163111st1.docx]

**Table S1: Resistance genes and Incompatibility groups of plasmids from 6 PacBio sequenced *Enterobacter* strains**

| **Plasmids** | **Plasmids-NCBI** | **Res finder** | **Replicon group** |
| --- | --- | --- | --- |
| p34399-A | p34399-43.500kb | *bla*_KPC-3_, *bla*_TEM-1A_ | pKPC_UVA01-like |
| p34399-B | p34399-106.698kb | *qnrS1* | IncF |
| p34399-C | p34399-121.660kb | - | FIB |
|  |  |  |  |
| p34977-A | p34977-5.006kb | - | ColE |
| p34977-B | p34977-43.621kb | *bla*_KPC-2_, *bla*_TEM-1B_ | pKPC_UVA01-like |
| p34977-C | p34977-263.138kb | *strB, strA, aadA2, aa(6')-llc, aph(3')-la, bla*_SHV-12_*, qnrB2, ere(A), sul1, sul1, sul1, sul2, dfrA18* | HI2, HI2A |
|  |  |  |  |
| p34978-A | p34978-2.725kb | - | ColE |
| p34978-B | p34978-4.938kb | - | ColE |
| p34978-C | p34978-5.413kb | - | ColE |
| p34978-D | p34978-13.828kb | - | ColE |
| p34978-E | p34978-70.092kb | - | L/M |
| p34978-F | p34978-139.941kb | *aac(6')-lb, aadA1, strB, strA*, *bla*_KPC-3_, *bla*_OXA-9_, *bla*_TEM-1A_, *aac(6')lb-cr, sul2, dfrA14* | FIA |
|  |  |  |  |
| p34983-A | p34983-43.621kb | *bla*_KPC-2_, *bla*_TEM-1B_ | pKPC_UVA01-like |
| p34983-B | p34983-59.134kb | *qnrB2, sul1, sul1, dfrB3* | N3 |
| p34983-C | p34983-328.905kb | - | HI1A, HI1B |
|  |  |  |  |
| p34998-A | p34998-4.921kb | - | ColE |
| p34998-B | p34998-53.129kb | - | N3 |
| p34998-C | p34998-106.409kb | *qnrS1* | IncF |
| p34998-D | p34998-210.894kb | *strA, strB*, *bla*_TEM-1B_, *sul2, tet(D), dfrA14* | FIB, FII |
| p34998-E | p34998-239.973kb | *aadA1, aac(3)-Vla, aph(3')-lc, aac(6')lb-cr,* *bla*_TEM-1A_, *bla*_KPC-4_, *bla*_OXA-1_, *aac(6')lb-cr, mph(A), catB3, arr-3, sul1, sul1* | A/C |
|  |  |  |  |
| p35734-A | p35734-8.452kb | - | ColE |
| p35734-B | p35734-109.753kb | *aac(6')-lb, aadA1*, *bla*_KPC-3_, *bla*_KPC-3_, *bla*_OXA-9_, *bla*_TEM-1A_, *aac(6')lb-cr, qnrB19* | pKPC_UVA01-like |
| p35734-C | p35734-141.404kb | - | A/C |
